# Hallmarks of Human Small Antral Follicle Development: Implications for Regulation of Ovarian Steroidogenesis and Selection of the Dominant Follicle

**DOI:** 10.3389/fendo.2017.00376

**Published:** 2018-01-12

**Authors:** Stine G. Kristensen, Linn S. Mamsen, Janni V. Jeppesen, Jane Alrø Bøtkjær, Susanne E. Pors, Tanni Borgbo, Erik Ernst, Kirsten T. Macklon, Claus Yding Andersen

**Affiliations:** ^1^Faculty of Health and Medical Sciences, Laboratory of Reproductive Biology, The Juliane Marie Centre for Women, Children and Reproduction, Copenhagen University Hospital, University of Copenhagen, Copenhagen, Denmark; ^2^The Fertility Clinic, Aarhus University Hospital, Aarhus, Denmark; ^3^The Fertility Clinic, Copenhagen University Hospital, Rigshospitalet, Copenhagen, Denmark

**Keywords:** human ovarian steroidogenesis, human small antral follicles, follicle-stimulating hormone, luteinizing hormone, granulosa cells, follicular fluid, inhibin-B, paracrine signaling

## Abstract

Regulation of human ovarian steroidogenesis differs from other species and precise knowledge on how human small antral follicles (hSAF) develop and acquire competence for continued growth and steroid output is still incomplete. The present study has characterized almost 1,000 normal hSAF collected in connection with cryopreservation of ovarian tissue for fertility preservation. The antral follicles (ranging from 3 to 13 mm) were generally aspirated from one ovary surgically removed during the natural cycle, and the follicular fluid (FF) and the granulosa cells (GC) were isolated and snap-frozen. In FF, the following hormones were measured: inhibin-B, inhibin-A, AMH, follistatin, PAPP-A, estradiol, progesterone, testosterone, and androstenedione. In GC, mRNA gene expressions using q-PCR were measured for the following genes: *FSHR, AMH, CYP19*, and *AR*. All samples in which one of the abovementioned parameters was measured were included, but typically multiple parameters were measured. Highly significant differences in concentration and follicular content in relation to follicular diameter were found for all measured hormones despite massive variability in-between follicles for any given diameter. The results demonstrate that profound changes take place in the hormonal microenvironment around follicular diameters of 8–11 mm corresponding to when follicular selection occurs. At this point, inhibin-B and inhibin-A showed distinct peaks concomitant with a significant reduction in both AMH protein and mRNA expression. Concentrations of inhibins, androgens, FSHR, and AR were intimately associated, and it is suggested that inhibin-B in combination with PAPP-A and thereby IGF2 activity exerts important paracrine signaling at follicular selection. At the same time upregulation of estradiol synthesis and *CYP19* mRNA expression increased steroid output profoundly. Furthermore, the highly significant association between *FSHR* and *AR* mRNA gene expression enforces important functions of androgens in follicular development. Collectively, these data reintroduce the understanding of the follicular phase as two parted in which regulation of steroidogenesis differs. The profound changes taking place around follicular selection highlight important paracrine actions of TGF-β family members and IGFs for securing dominance of the selected follicle.

## Introduction

Follicles are the functional units of the ovaries. Each follicle consists of a single oocyte surrounded by somatic granulosa cells (GC) covering the internal surface of the basal membrane that encompasses follicles and prevents vessels from entering. Early in development, follicles recruit theca cells immediately adjacent to the basal membrane encircling the follicle. The theca cells become highly vascularized and secure transport of hormones including gonadotrophins to and from both the follicle and the theca cells. When human follicles reach a diameter of around 200–300 µm a fluid-filled antral cavity starts to form, which contains the follicular fluid (FF). The FF consists of filtrations or transudates from circulation that penetrate the basal membrane and of GC secretions including a number of steroid and peptide hormones (1). The composition of hormones in FF is very dynamic in relation to developmental stage and very different from conditions found in circulation (2–4). For instance, steroidogenesis is very prominent throughout most of follicular development with synthesis of androgens in the theca cells by stimulation with luteinizing hormone (LH), which to a high degree is converted to estrogens in GC *via* stimulation with follicle-stimulating hormone (FSH), illustrating the two-cell, two-gonadotropin concept. As the follicle matures, the concentration of steroids such as estradiol and progesterone increases and reaches levels 1–10,000-fold higher than in circulation. In addition, several peptide hormones are specifically synthesized by the GC including AMH, inhibin-A, and inhibin-B. They also accumulate in FF often with concentrations more than 1,000 times higher than in circulation (4, 5). Growth and development of human small antral follicles (hSAF) are affected by these and other hormones, and a number of both autocrine and paracrine effects are dependent on the very different and local concentrations present in FF and in theca cells (6–8). It has been suggested that inhibin-B in addition to attenuate pituitary FSH secretion also plays an important paracrine effect in augmenting theca cell androgen production in synergy with LH (9–12). These fine-tuned local mechanisms are likely to exert an important function in the process of selecting the dominant follicle that takes place in each individual menstrual cycle (5). Almost four decades ago, it was realized that each individual follicle possesses a unique hormonal fingerprint and that the microenvironment in the follicle to a large extent determines its further developmental capacity (13). Furthermore, the dynamic changes in FF in relation to the developmental stage of the human follicles are massive, probably reflecting the variable hormonal and nutritional requirement of the developing follicle and the enclosed oocyte (14). Although FF from large preovulatory follicles, especially as collected in connection with IVF treatment, has received a lot of attention scientifically, normal hSAF have only to a limited degree been studied mainly because of unavailability. However, this has changed in recent years because women in connection with fertility preservation often have one entire ovary excised for cryopreservation (15). The hSAF do not sustain freezing and are normally discarded. During the last 10 years, we have accumulated a large number of hSAF including FF and corresponding GC.

The aim of the present study was to characterize the hallmarks of hormonal and genetic markers of normal hSAF, spanning in follicular diameter from 3 to 13 mm. This cohort of follicles thereby constitutes the follicular starting point for ovarian stimulation with exogenous hormones as used in connection with infertility treatment.

## Materials and Methods

All follicles were collected from surplus ovarian tissue from women undergoing fertility preservation, having ovarian tissue cryopreserved at the Laboratory of Reproductive Biology, Rigshospitalet, Denmark. The fertility preservation procedure normally involved excision of one entire ovary from which individual visible antral follicles were aspirated with a 23G needle attached to a syringe. The fluid was centrifuged, the FF aspirated and immediately stored at −80°C or snap frozen in liquid nitrogen. The diameter of the follicles was calculated based on the aspirated volume. In some cases, the GC was also collected and snap frozen in liquid nitrogen.

Collection of follicles has taken place during the last 15 years, and parts of the present material of both FF and GC have been used in several individual studies (16–26). Included in the present study is any follicle on which at least one of the reported parameters has been measured. Excluded from the analysis are girls below the age of 16 years and those who had a diagnosis for fertility preservation that included a disease of the ovary (e.g., ovarian cancer). The diagnoses of the women included were as follows: cancer mammae: 133; Md. Hodgkin: 41; non-Hodgkin and other lymphoma: 20; Ewing sarcoma and other sarcoma: 14; cervix: 13; leukemia: 13; cancer cerebrum: 12; colon cancer: 7; autoimmune diseases: 5; others: 28. The collection of FF had no effect on the fertility preservation procedure. In all cases, the ovary had overall gross normal appearance.

**Figure 1:**
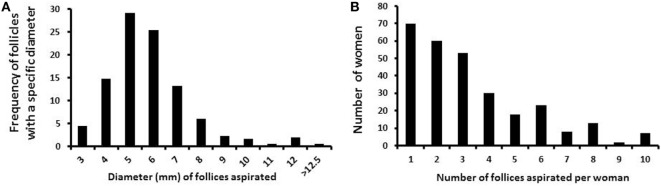
Number and diameter of follicles collected per woman. **(A)** Bars depict the frequency of small antral follicles in relation to the specific diameters. **(B)** Bars depict the number of small antral follicles collected per woman.

In total, 958 follicles were collected from 286 women, with a mean age of 28 years (ranging from 16 to 43 years). The number of follicles collected from each woman ranged from 1 to 13 (mean 3.3 follicles/patient; median 3.0). The number of women in relation to the number of follicles aspirated is given in Figure 1A. Follicle diameters ranged from 3 to 13 mm, as calculated from the aspirated volume, assuming a spherical structure of the follicle. The frequency of follicles aspirated with a specific diameter (e.g., 5 mm follicles compromise follicles with 4.5–5.5 mm in diameter) from the cohort of follicles is depicted in Figure 1B.

The hormone milieu in hSAF has been shown to be independent of circulating levels of gonadotropins and has no major impact on the serum hormone levels, thus serum hormone levels were not included in this study (22, 26, 27). The follicles were collected at various times during the menstrual cycle, as the hormone levels of hSAF remain constant throughout the menstrual cycle (23, 27, 28).

### Hormone Measurements

The concentrations of AMH, inhibin-A, and inhibin-B were measured using commercially available ELISA assays. AMH was measured using the Ultra-Sensitive AMH/MIS ELISA kit (Al-105-i, Ansh Labs, Webster, TX, USA), Inhibin-A ELISA kit (AL-123-i, Ansh Labs, Webster, TX, USA), and Inhibin-B ELISA kit (AL-107-i Ansh Labs, Webster, TX, USA) according to the manufacturer’s protocol, with an appropriate dilution of the FF samples using the supplied assay buffer. Estradiol, progesterone, androstenedione, and testosterone were initially measured using commercially available RIA kits (DSL-43100, DSL-3400, DSL-3800, and DSL-4000; DSL, Webster, TX, USA). However, during the course of sample collection, the RIA assays became unavailable, and it was necessary to switch to ELISA assays (NovaTec Immundiagnostica, Dietzenbach, Germany, DNOV 002, 003, 006, and 008, respectively). Due to limited sample material, it was not possible to reanalyze the FF with ELISA kits. Based on highly significant linear correlations between the results from the two types of assays (correlation coefficients: estradiol *r* = 0.99, progesterone *r* = 0.93, testosterone *r* = 0.91, and androstenedione *r* = 0.91), mathematical conversion of values to the ELISA standard was performed as previously published (21). The remaining follicles were analyzed using NovaTec ELISA assays, according to the manufacturer’s protocol, using in-house prepared steroid-free serum for FF dilution.

### RNA Purification

From individual GC samples, total RNA was purified using a combination of Tri Reagent (Sigma-Aldrich, St. Louis, MO, USA) and the RNeasy Mini Kit (Qiagen, Hilden, Germany). For each sample, the quality of the purified RNA was analyzed using an Agilent 2100 Bioanalyzer and an RNA 6000 Pico LabChip (RNA 6000 Pico assay kit, Agilent Technologies, Waldbronn, Germany). The RNA integrity number should be higher than the internal threshold value in order to qualify samples for the study. Total RNA quantity in each sample was measured using a Beckman Coulter Du730 life science UV/vis spectrophotometer.

### cDNA Synthesis and qPCR Analysis

First-strand cDNA was synthesized using the High Capacity cDNA Reverse Transcription Kit (Applied Biosystems, Carlsbad, CA, USA). Briefly, a master mix containing 2.0 µl 10× RT Buffer, 0.8 µL 25× dNTP Mix (100 mM), 2.0 µl 10× RT Random Primers, 1.0 µl MultiScribe™ Reverse Transcriptase (50 U/μl), 0.5 µl RNase inhibitor, and 4.7 µl nuclease-free (DEPC) water was prepared for each 20 µl reaction. 11 µl master mix was added to 9 µl of total RNA. Samples were centrifuged briefly at 12,000 × *g* and then incubated at room temperature for 10 min, followed by 37°C for 2 h and finally, 85°C for 5 s.

Gene expression levels were evaluated by relative quantification-PCR analysis using TaqMan^®^ technology (Applied Biosystems) and the LightCycler 480 qPCR instrument (Roche, Copenhagen, Denmark). Predesigned AMH, FSHR, AR, and CYP19a1 TaqMan^®^ Gene Expression Assays, as well as the Endogenous Control Assays for human β-actin and glyceraldehyde 3-phosphatdehydrogenase (GAPDH) were purchased from Applied Biosystems [Assay id no.: β-actin: #4326315E, GAPDH: #4333764F, AMH: #Hs00174915_m1, FSH-R: #Hs00174865_m1, AR: #Hs00171172_m1, and Cyp19a1 (aromatase): #Hs00903413]. Data were quantified according to the comparative CT method (LightCycler480 Software, Roche); expression levels are presented as 2-ΔCT normalized to GAPDH (20).

### Statistical Analysis

Statistical analysis was performed using GraphPad Prism 6.07 program (GraphPad Software, Inc., CA, USA). One-way analysis of variance (ANOVA) was used to determine the significant differences in hormone levels and follicular content in relation to the follicular diameter of hSAFs, and mRNA gene expression of GC-specific substances in hSAF in relation to follicular diameter. Spearman’s rank correlation was used to evaluate correlations between various hormones in FF and between genes expressed in GC. A level of *P* < 0.05 was considered statistically significant.

## Results

The number of follicles aspirated per woman varied from 1 to 13, with the vast majority having one or two follicles aspirated (Figure 1A). The diameter of follicles aspirated from women above the age of 15 years is depicted in Figure 1B. Follicles with diameters from 4 to 7 mm account for more than 80% of the all analyzed follicles.

### Follicular Content and Concentrations of Peptide and Steroid Hormones

**Figure 2:**
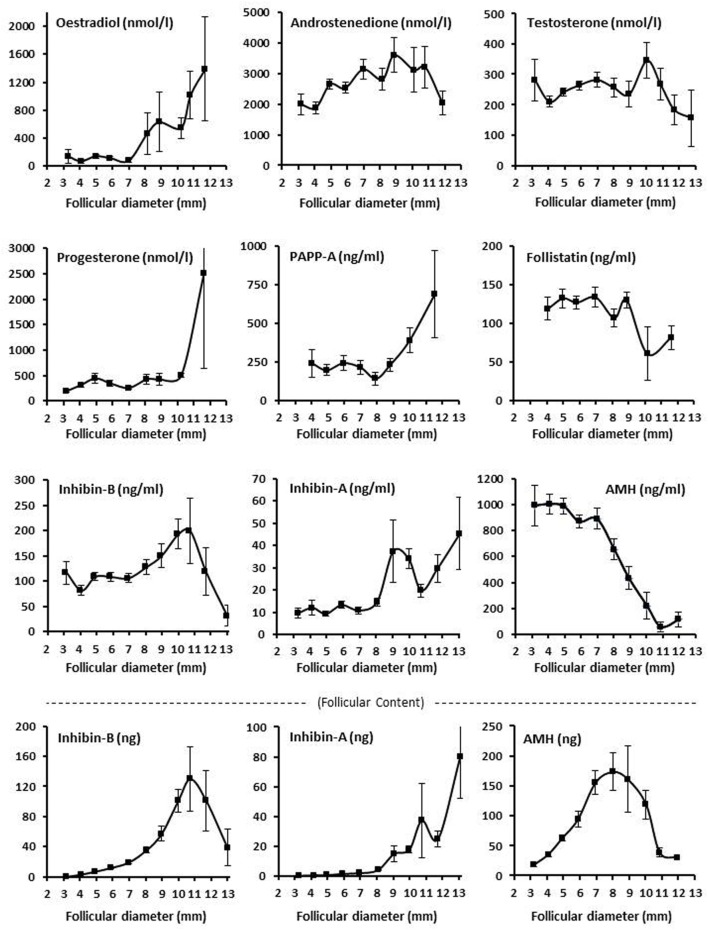
Intrafollicular content and concentrations of peptide and steroid hormones. Graphs show the follicular fluid concentrations of estradiol, progesterone, androstenedione, testosterone, inhibin B, inhibin A, AMH, PAPP-A, and follistatin, and follicular content in nanograms (bottom panel) of inhibin B, inhibin A, and AMH according to follicular diameter. Values are presented as mean ± SEM. Statistically significant differences were observed in intrafollicular hormone levels of estradiol [analysis of variance (ANOVA); *N* = 264; *P* < 0.001], androstenedione (ANOVA; *N* = 276; *P* < 0.03), progesterone (ANOVA; *N* = 267; *P* < 0.003), PAPPA (ANOVA; *N* = 96; *P* < 0.03), inhibin-B (ANOVA; *N* = 627; *P* < 0.002), inhibin-A (ANOVA; *N* = 258; *P* < 0.001), AMH (ANOVA; *N* = 655; *P* < 0.001), but not testosterone (ANOVA; *N* = 566; *P* > 0.10) and follistatin (ANOVA; *N* = 148; *P* > 0.10). Regarding follicular content (bottom panel), the intrafollicular contents of inhibin-B (ANOVA; *N* = 611; *P* < 0.001), inhibin-A (ANOVA; *N* = 258; *P* < 0.001), and AMH (ANOVA; *N* = 654; *P* < 0.001) were observed to be significantly different in relation to follicular diameter. Specific numbers for the analyzed follicle groups divided according to follicular diameter are shown in Tables S1 and S2 in Supplementary Material.

The intrafollicular concentration of inhibin-B showed a highly significant variation with follicular diameter showing peak concentrations around diameters of 9–11 mm. The follicular content of inhibin-B also showed a highly significant variation with follicular diameter with a peak at 11 mm and high flanking values in 10 and 12 mm follicles (Figure 2; Tables S1 and S2 in Supplementary Material).

Inhibin-A exhibited a peak at around 9–10 mm similar to inhibin-B, but inhibin-A was present in concentrations comprising around 8–20% of that of inhibin-B with a relative concentration of inhibin-A increasing as the follicular diameter increased (3–11 mm). When the diameter exceeded 13 mm, the picture reversed and the concentration and content of inhibin-A exceeded that of inhibin-B (Figure 2; Tables S1 and S2 in Supplementary Material).

Both the concentration and the content of AMH showed a highly significant association with the follicular diameter with peak values at around a diameter of 8 mm (Figure 2; Tables S1 and S2 in Supplementary Material). Noteworthy is the follicular content of inhibin-B, inhibin-A, and AMH (calculated in nanograms), which was in a similar order of magnitude comprising around 100–200 ng at peak levels with a little less inhibin-A. Despite the relative smooth curves based on the mean levels and mean content of the follicles, the variability in follicles with a given diameter was very high (Tables S1 and S2 in Supplementary Material). There was a highly significant negative association between the FF concentrations of AMH and inhibin-B irrespective of follicular diameter (Spearman rank sum test: *N* = 538; *R* = 0.46; *P* < 0.00001).

**Figure 3:**
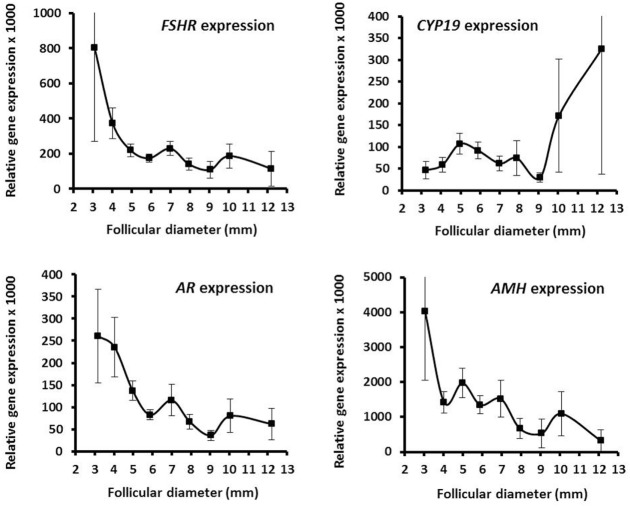
Granulosa cell expression of selected genes in human small antral follicles. Graphs show relative expression levels of *FSHR, CYP19a1, AR*, and *AMH* in relation to follicular diameter. Values are presented as mean ± SEM. Statistically significant differences were observed in the mRNA expression levels of *FSHR* [analysis of variance (ANOVA); *N* = 315; *P* < 0.002] and *AR* (ANOVA; *N* = 316; *P* < 0.03), but not for *CYP19a1* (ANOVA; *N* = 252; *P* > 0.10) and *AMH* (ANOVA; *N* = 277; *P* > 0.10). Specific numbers for the analyzed follicle groups divided according to follicular diameter are shown in Table S3 in Supplementary Material.

The intrafollicular concentrations of the four evaluated steroids showed different associations with the follicular diameter. Androstenedione and testosterone were both relatively constant, but testosterone showed a peak at a follicular diameter of 10 mm (Figure 2). In contrast, estradiol starts to increase at around 8–10 mm (Figure 2), coinciding with a reduction in intrafollicular AMH concentrations and GC expression (Figures 2 and 3). Progesterone remained relatively constant but ended with a steep increase in concentration at a follicular diameter of 11–12 mm (Figure 2). The association between the follicular diameter and the intrafollicular concentrations of follistatin and PAPP-A is also shown in Figure 2. PAPP-A showed a steep increase in the concentration starting with a follicular diameter of 8 mm, whereas follistatin was relatively constant with a drop in concentration at around 8–10 mm.

### Gene Expression Profiles of Selected Genes

The mRNA expression profiles of GC showed that *FSHR* expression is highly dependent on the follicular diameter and decreases with increasing diameter (Figure 3). This contrasts *CYP19* mRNA expression that in parallel to the concentration of estradiol starts to increase at diameters of 8–10 mm (Figure 3). The *AR* mRNA expression in relation to follicular diameter showed a decreasing expression as the follicular diameter increase in close association with the *FSHR* expression (Figure 3). There was a highly significant association between mRNA expression of *AR* and *FSHR* (Spearman rank sum test: *N* = 310; *R* = 0.77; *P* < 0.00001). The *AMH* expression decreased with increasing follicular diameter as expected (Figure 3). As for the substances present in FF, the variability of the gene expression was massive in between individual follicles with similar diameter (Table S3 in Supplementary Material).

## Discussion

**Figure 4:**
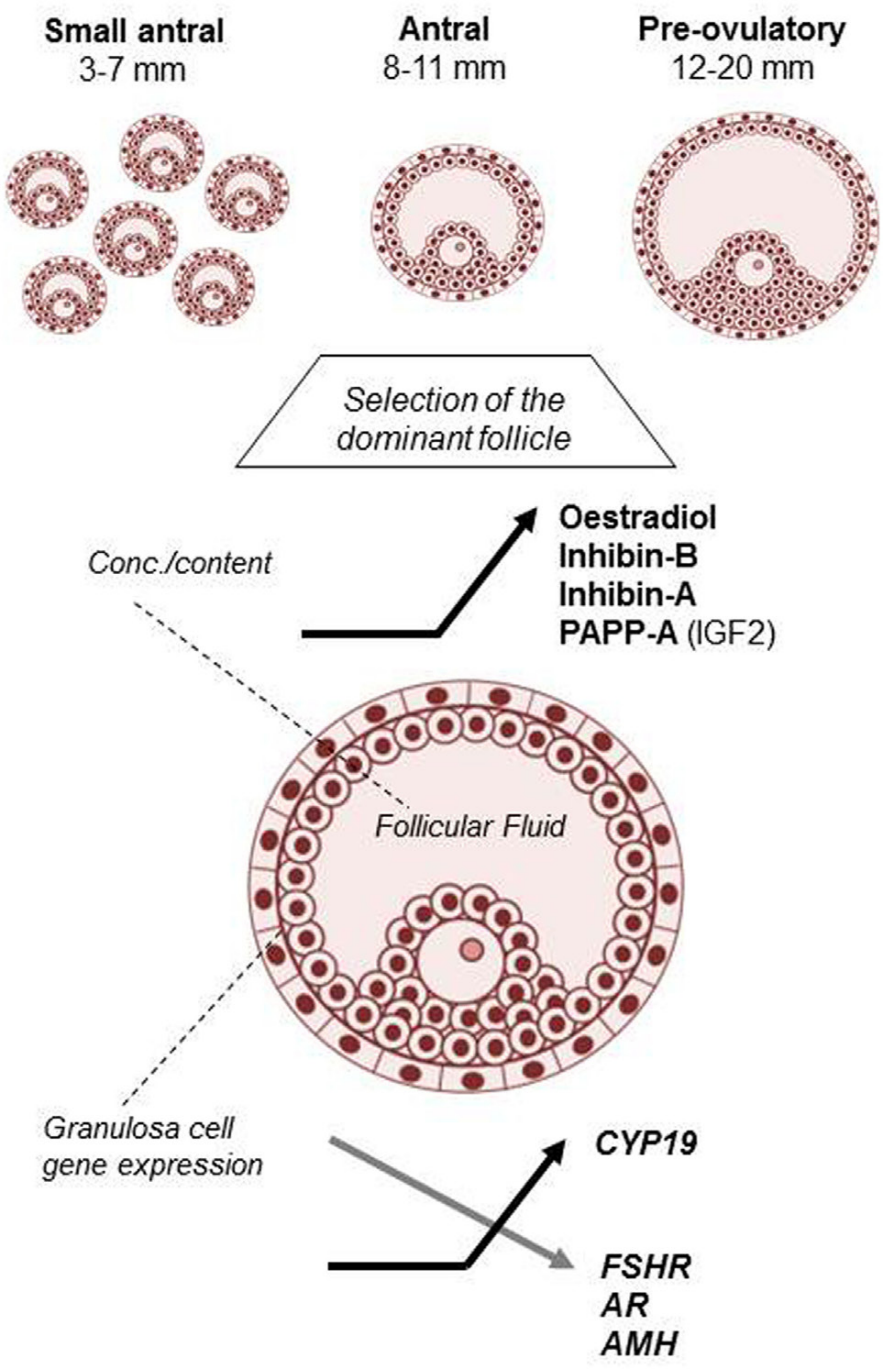
Hallmarks of human small antral follicle development: implications for regulation of ovarian steroidogenesis and selection of the dominant follicle.

Including data from almost 1,000 normal hSAF, the present study documented profound changes taking place in the average concentrations of multiple key FF hormones and GC-expressed genes in relation to follicular development. It is especially noticeable that for all the secreted hormones, major alterations in the intrafollicular concentrations emerge with diameters from around 8 to 11 mm (Figure 4). This corresponds to the follicular stage in which selection of the dominant follicle takes place in each individual menstrual cycle and demonstrates the dramatic changes that occur during the transition of follicles from the recruitable stage to the selected follicle destined to ovulate with a pronounced shift in steroidogenesis resulting in a massive synthesis of estradiol/CYP19 (aromatase) and progesterone. Overall, this enforces the notion introduced by Yen and Jaffe (29) almost half a century ago, hormonal regulation of the follicular phase of the menstrual cycle differs in two periods during a first half lasting to around cycle day 7 and a second half starting from the day of follicle selection and until ovulation.

Furthermore, the current data not only corroborate and extend that regulation of ovarian steroidogenesis during the follicular phase of the menstrual cycle is governed by FSH and LH but also highlight that local TGF-β growth factors exert important paracrine and autocrine functions that act to secure the development of one competent follicle selected for ovulation per cycle.

The present study demonstrates that intrafollicular concentrations of estradiol start to rise rapidly in the window of follicular selection (i.e., 8–11 mm) paralleled by concomitant increase in *CYP19* mRNA expression. Several mechanisms are probably involved in this strong upregulation. First, inhibins become synthesized as a result of FSH stimulation. Thus, the follicle that secretes high amounts of inhibins probably also possesses high FSHR expression. So not only does FSH secure substrate for aromatization *via* inhibin and subsequent androgen synthesis but does also stimulate aromatase and LHR expression, which are essential for estradiol production during the second half of the follicular phase (5). Despite the high levels of FSH in first half of the follicular phase aromatase expression and estradiol production remain relatively low (30) as the FF data of the present study also suggest. This appears to be an effect of AMH which in the very high concentrations present in hSAF exert a negative effect on estradiol production (18, 22, 31). At follicular selection, the dominant follicle acquires strong aromatase expression and upregulate estradiol production, which to a large extent appears to be driven by decreasing levels of AMH (attenuating) and increasing levels of inhibins (augmenting). In this scenario, AMH and inhibins could be expected to be inversely associated, which indeed is the case in the present study and confirms an earlier study (22). This suggests that both AMH and inhibin-B are intimately associated and both involved in the regulation of follicular estradiol synthesis.

In addition, IGF2 is also likely to play an important role in the regulation of estradiol synthesis by the selected follicle. The present study showed that the intrafollicular concentration of PAPP-A increased almost threefold in follicles with diameters increasing from 8 to 12 mm. Again, assuming that PAPP-A expression reflects IGF bioactivity (32–36), it is noteworthy that PAPP-A expression moves from the theca cells to the GC as the follicles become dominant (24, 34–36). The increased IGF activity may serve two purposes: first, the exceptional high proliferation of GC during the second half of the follicular phase probably requires a strong local signal especially since the FSHR expression becomes downregulated (21). Second, IGF2 on its own stimulates human GC estradiol synthesis (37–40), which has recently been shown with highly significant associations between PAPP-A activity in FF and estradiol concentrations in FF from different-sized follicles (24, 34).

It is surprising that *FSHR* expression per cell declines with follicular diameter and is significantly reduced at follicular selection during the natural menstrual cycle, confirming an earlier study (21). When GC from preovulatory follicles prior to ovulation induction were challenged by similar concentrations of FSH and LH, aromatase *in vitro* induction was clearly biggest after stimulation with LH, and in connection with ovarian stimulation for infertility treatment it has been shown that just a modest addition of LH-like activity results in a pronounced augmentation of estradiol. Collectively suggesting that LH probably also plays a prominent role in stimulation of aromatase expression. Furthermore, it is likely that there is a redundancy between FSH and LH in induction of aromatase expression and that ovarian stimulation with exogenous FSH preparations overcomes a reduction in *FSHR* expression. Furthermore, as the present results are expressed as FSHR expression per cell, the collective follicular FSHR number may still increase because of the hectic proliferation of GC as the follicle enlarges.

The inhibitory effect of inhibins in concentrations of picograms per milliliter on pituitary FSH release is well recognized, but the importance of the simultaneous paracrine effect of inhibins in synergy with LH on theca cell androgen synthesis continues to be unrevealed in human ovaries. The intrafollicular concentration of inhibin-B plus inhibin-A remained relatively constant around 100–120 ng/ml in follicles with diameters of 3–7 mm but doubled to 200–220 ng/ml and showed a peak in follicles with diameters of 9–10 mm. Previous *in vitro* studies of human theca cells showed that inhibins act in synergy with LH to upregulate synthesis of androstenedione (8–11, 41–45). The maximal concentration of inhibins tested in these studies was 100 ng/ml. Indeed, the collective intrafollicular concentrations reach at least twice this concentration and make it conceivable that the theca cells actually are exposed to such high concentrations *in vivo*. Obviously, theca produced androstenedione requires further metabolism to testosterone and to diffuse to the follicular compartment to exert a true androgenic effect. In this context, it is noteworthy that intrafollicular concentrations of testosterone in parallel to intrafollicular inhibins also peak in follicles with diameters of around 9–11 mm in line with recent results showing strong significant associations between inhibin-B and androstenedione and testosterone in hSAFs (5). The intrafollicular testosterone concentrations exert a stimulatory effect on the GC expression of *FSHR* (19, 46, 47) and subsequently expression of *LHR*. Androgens act through the AR, and the notion is enforced by the highly significant association between *FSHR* and *AR* mRNA levels in GC from hSAFs of the present study (19). Thereby, the selected follicle on one side secures not only a sufficient substrate for a high estrogen production but also a sufficient FSHR expression, which in light of declining FSH levels is required to secure sufficient LHR expression to sustain ovulation. In fact, the shape of the *FSHR* and *AR* mRNA expression profile in relation to follicular diameter is almost superimposable and suggests a close interrelationship.

It has also been reported that in humans especially IGF2 augment theca cell-derived androgen production in synergy with LH [human (48–50), primate (51), and rats (11, 52, 53)]. It has previously been difficult to account for the biological activity of IGFs because there are several different binding proteins (i.e., IGFBP 1–6) inactivating IGF activity, proteases that specifically cleave different IGFBPs to release bound IGFs (i.e., PAPP-A and PAPP-A2), and furthermore specific proteins that inactivate the proteolytic activity of PAPP-A (i.e., stanniocalcin 1 and 2) (32, 34). On top of this complicated system for regulating IGF bioactivity, it appears that the presence of PAPP-A covalently linked to the cell membrane secures a local microenvironment where IGFs released after specific cleavage by PAPP-A have a possibility to activate IGF receptors and cause signal transduction (32). Collectively, however, several studies have now shown that concentrations of PAPP-A appear to be a good surrogate marker for the bioactivity of IGFs (32, 34). It has been shown that PAPP-A becomes expressed in the theca cells of hSAF moving to the GC as the follicle develops further (24). These results suggest a local production of IGFs that corroborate *in vitro* studies (11, 48–52) that showed a synergistic effect of LH and IGFs on theca cell androgen production.

Collectively, these new data support the original hypothesis proposed by Hillier and coworkers in their landmark studies on human tissue in the early 1990s and place especially inhibin-B and IGF2 in a physiological context as important paracrine regulators augmenting steroid output by especially the selected follicle in the second half of the human follicular phase.

Both inhibins and IGFs themselves can independently stimulate androgen production in synergy with LH, but it appears that they utilize separate mechanisms. Inhibins are capable of enhancing the combined maximal stimulatory effect of LH and IGF1 with an up to 10-fold higher androgen output in human theca cells *in vitro* (12, 43).

While inhibins augment theca cell androgen production, activins, in contrast, have been shown to exert the opposite effect and reduce androgen output *via* downregulating expression of CYP17 and 3β-hydroxy steroid dehydrogenase [human (12, 54), rat (9), bovine (55, 56), sheep (42, 44, 57)]. No specific receptors for inhibins have been identified and no corresponding second messenger signaling pathway system identified. The affinity of activin for the activin type II receptor is around 10-fold higher than that of inhibins (58), but by binding to the membrane bound β-glycan, inhibins augment interaction with the activin type II receptor and enhance its effect as an activin antagonist (6). In this context, it is interesting to notice that FF concentrations of inhibin-A start to surpass those of inhibin-B when follicles exceed a diameter of around 13–14 mm. In circulation, this is only observed close to ovulation when the effect of massive inhibin-B production in connection with selection is being reduced and suggests that theca cell androgen production is maintained at a high level until ovulation.

Taken together, regulation of androgen production in hSAF is likely to involve an upregulation of LHR number *via* locally expressed PAPP-A, which leads to enhanced IGF2 activity that acts on the IGFR being expressed on theca cells (59). Independent of the IGF2 action, inhibins in FF of hSAF being present in high concentrations reduce a negative impact of activins and enhance the 17-hydroxylase/C_17–20_ lyase activity that further augments the androgen producing capacity.

In this context, it is noteworthy that LHR numbers have been shown to be a major determinant for hCG-induced steroidogenic output from cells during culture highlighting that LHR density is of importance for LH action (60). Therefore, the follicle where the surrounding theca cells respond with highest production of androgen is most likely to become the selected follicle destined to ovulate as recently described (5). The present data emphasize the exquisite sensitivity of theca cells to LH stimulation in combination with inhibins, IGFs, and potentially other locally produced growth factors in the regulation of ovarian steroidogenesis and function.

Collectively, a local amplification of thecal androgen production by inhibins and IGF2 suggests a mechanism through which follicular dominance is acquired and maintained in human reproductive cycles.

A major limitation of the present study is the inability to distinguish between healthy follicles and follicles which have started to undergo atresia. A fraction of the follicles in this study have already become atretic to some degree (61), but will be saved by administration of exogenous FSH preparations. It is well-known that oocytes from a cohort of follicles as a result of exogenous FSH stimulation may result in several children pointing at a clear clinical interest. The degree of atresia is likely to affect steroidogenesis and to what extent this affects the outcome measures as reported in the present study is unknown. This may reflect that the FF concentrations as reported in the present study show a massive range, often more than three orders of magnitude from one follicle to the other, which, however, may not be linked to atresia but rather reflect other factors. Obviously, there is a whole range of methods to detect the rate of atresia, but the often very limited number of GC from each individual follicle does not allow for detailed analysis.

The present study has only addressed a limited number of albeit prominent TGF-β growth factors and their presence in FF from hSAF and their potential effect on ovarian steroidogenesis. Indeed, other TGF-β growth factors have been reported to affect ovarian steroidogenesis, and it is likely that regulation involves a number of other factors such as BMP 4, 6, and 7 (42, 54, 62, 63).

It is interesting to notice that a number of the mechanisms proposed to regulate ovarian steroidogenesis involve release of biological active substances in the vicinity of the cell membrane. Covalently coupled PAPP-A appears to cleave IGFBP to release IGFs close to the membrane bound receptor (33), and locally high concentrations of inhibins can attenuate even locally produced activin from interaction with its receptor are examples that may improve our understanding of how the fine-tuned hormonal regulation of ovarian steroidogenesis takes place *in vivo*.

In conclusion, the present study suggests that regulation of human ovarian steroidogenesis during the follicular phase differs between the first part of the follicular phase in which recruitment takes place and the second half where development of the dominant follicle concomitant with a massive output of estradiol. Especially with follicular diameters of around 8–11 mm, regulation of steroid production change profoundly with significant impact from paracrine and autocrine regulation of TGF-β growth factors especially inhibins and activins that together with IGF2 *via* PAPP-A modulate and fine-tune the effects of FSH and LH in order to secure the development of one dominant follicle in each menstrual cycle. The pronounced effects of inhibin-B are noticeable acting both to reduce pituitary output of FSH, but also as a follicle derived signal to augment androgen production locally by the theca cells, which in turn upregulate *FSHR* mRNA expression that enables the dominant follicle to respond to the decreasing levels of FSH by having aromatase and *LHR* mRNA expression upregulated.

## Ethics Statement

This study was carried out in accordance with the recommendations of the ethical committee of the municipalities of Copenhagen and Frederiksberg with written informed consent from all subjects. All subjects gave written informed consent in accordance with the Declaration of Helsinki. The protocol was approved by the ethical committee of the municipalities of Copenhagen and Frederiksberg (journal number; H-2-2011-044).

## Author Contributions

CA and SK designed the study and wrote the manuscript. KM and EE enrolled patients for the study. SK, JJ, and SP collected follicular fluids and GCs for the study. TB, JB, and JJ performed the gene expression analysis and ELISA/RIA analysis.

## Conflict of Interest Statement

The authors declare that the research was conducted in the absence of any commercial or financial relationships that could be construed as a potential conflict of interest.
