# Supplementary material for: Hallmarks of Human Small Antral Follicle Development: Implications for Regulation of Ovarian Steroidogenesis and Selection of the Dominant Follicle
Source: Front Endocrinol (Lausanne). 2018 Jan 12;8:376. doi: 10.3389/fendo.2017.00376 (PMC5770355; doi:10.3389/fendo.2017.00376)
Supplement: Supplementary file 1 [file Table_1.PDF]

Supplemental table 1. Hormonal characteristics of follicular fluid concentrations of human small antral follicles in relations to follicular diameter.

		FOLLICULAR DIAMETER (MM)											
HORMONE		<3.5	3.5-4.5	4.5-5.5	5.5-6.5	6.5-7.5	7.5-8.5	8.5-9.5	9.5-10.5	10.5-11.5	11.5-12.5	>12.5	SUM No. /P-value
Inhibin-B (ng/ml)	N	17	77	174	162	87	51	24	16	5	9	5	627
	M±SEM	116 ±23	81 ±10	109 ±8	108 ±8	106 ±9	128 ±14	150 ±24	193 ±29	199 ±65	118 ±47	31 ±21	P<0.002
	Range	18-395	3-421	10-500	11-500	11-500	12-447	16-500	10-361	67-423	20-360	15-108	
Inhibin-A (ng/ml)	N	6	23	60	59	32	28	18	14	4	9	5	258
	M±SEM	10 ±2	12 ±3	9 ±1	13 ±2	11 ±2	15 ±2	37 ±14	34 ±4	20 ±3	30 ±6	45 ±16	P<0.001
	Range	2-15	3-75	2-50	2-63	3-48	3-47	4-257	6-57	12-24	5-64	22-109	
AMH (ng/ml)	N	24	98	178	178	89	45	17	14	3	9		655
	M±SEM	993 ±155	1007 ±77	990 ±60	871 ±50	893 ±81	656 ±78	434 ±85	220 ±102	59 ±39	115 ±54		P<0.001
	Range	10-2638	15-3918	4-4440	21-3357	16-3516	10-2086	24-1182	14-1468	8-136	12-453		
Oestradiol (nmol/l)	N	3	35	80	83	31	13	7	4	2	6		264
	M±SEM	139 ±101	67 ±26	139 ±31	112 ±26	75 ±23	461 ±301	627 ±427	545 ±148	1016 ±338	1392 ±748		P<0.001
	Range	4-338	4-835	1-1344	3-1354	3-552	5-3867	12-3094	297-890	677-1354	23-3925		
Progeste-rone (nmol/l)	N	7	36	80	81	31	12	7	4	9			267
	M±SEM	195 ±31	314 ±38	445 ±95	349 ±54	257 ±27	429 ±100	426 ±116	499 ±45	2502 ±1865			P<0.003
	Range	80-332	66-951	6-7340	25-4333	16-607	70-1168	90-970	373-583	119-17392			
Androstene-dione (nmol/l)	N	9	40	79	84	31	12	7	5	2	7		276
	M±SEM	2000 ±333	1886 ±189	2648 ±162	2530 ±182	3142 ±326	2809 ±360	3606 ±577	3116 ±726	3216 ±677	2038 ±383		P<0.03
	Range	705-3810	99-4655	295-6984	70-6928	677-6963	911-5360	1467-5996	1320-5685	2539-3894	1044-3719		
Testoste-rone (nmol/l)	N	19	89	158	152	72	35	14	12	3	9	3	566
	M±SEM	281 ±69	210 ±18	242 ±13	264 ±15	281 ±24	256 ±30	234 ±42	345 ±58	267 ±52	184 ±49	156 ±93	P>0.10
	Range	89-1409	11-902	33-757	16-860	27-1133	15-701	59-680	48-649	163-329	59-419	36-340	

Data is mean ±SEM. P-value <0.05 considered significant (ANOVA test).
