# Supplementary material for: Hallmarks of Human Small Antral Follicle Development: Implications for Regulation of Ovarian Steroidogenesis and Selection of the Dominant Follicle
Source: Front Endocrinol (Lausanne). 2018 Jan 12;8:376. doi: 10.3389/fendo.2017.00376 (PMC5770355; doi:10.3389/fendo.2017.00376)
Supplement: Supplementary file 2 [file Table_2.PDF]

Supplemental table 2. Hormonal content in follicular fluid from human small antral follicles in relations to follicular diameter.

		FOLLICULAR DIAMETER (MM)											
HORMONE		<3.5	3.5-4.5	4.5-5.5	5.5-6.5	6.5-7.5	7.5-8.5	8.5-9.5	9.5-10.5	10.5-11.5	11.5-12.5	>12.5	SUM No. /P-value
Inhibin-B (ng)	N	77	174	162	87	52	24	16	5	9	5	77	611
	M±SEM	3 ±0.4	7 ±1	12 ±1	19 ±2	35 ±4	57 ±9	101 ±15	130 ±42	101 ±40	39 ±24	3 ±1	P<0.001
	Range	2-17	2-33	1-55	2-75	2-134	6-190	1-215	42-271	2-324	2-129	2-17	
Inhibin-A (ng)	N	6	23	60	59	32	28	18	14	5	9	4	258
	M±SEM	0.2 ±0.0	0.5 ±0.1	0.6 ±0.1	1.5 ±0.2	1.9 ±0.3	4.0 ±0.5	14.8 ±5.4	17.7 ±2.4	37.2 ±24.7	25.0 ±5.4	80 ±28	P<0.001
	Range	0.1-0.3	0.1-3.0	0.1-4.3	0.2-7.6	0.5-9.0	0.8-12.6	1.5-97.7	2.8-34.4	8.3-136.0	4.2-57.3	26-135	
AMH (ng)	N	24	98	178	178	89	46	17	14	3	7		654
	M±SEM	18 ±3	35 ±3	63 ±4	94 ±6	156 ±14	173 ±20	161 ±32	119 ±56	38 ±24	30 ±8		P<0.001
	Range	0.1- 46	1-120	1-271	3-336	3-563	3-554	10-431	7-807	6-84	10-65		
Oestradiol (nmol)	N	3	35	80	83	31	14	7	3	2	7		265
	M±SEM	2 ±1.5	2 ±0.8	9 ±2	12 ±3	13 ±4	140 ±84	229 ±158	235 ±56	684 ±264	1015 ±560		P<0.001
	Range	0.1-5	0.1-25	0.1-108	0.4-190	1-94	2-1160	4-1145	178-348	420-948	20-3218		
Progeste-rone (nmol)	N	7	36	80	81	31	13	7	4	8			277
	M±SEM	3 ±0.4	11 ±1.4	28 ±6	36 ±6	46 ±5	120 ±26	154 ±41	279 ±26	519 ±115			P<0.001
	Range	1.6-5,0	2.0-38	0.3-440	3-433	4-134	20-292	36-340	224-350	129-1089			
Androstene-dione (nmol)	N	9	40	79	84	31	13	7	5	2	7		276
	M±SEM	34 ±6	68 ±7	170 ±11	269 ±20	561 ±61	819 ±99	1324 ±212	1697 ±449	2096 ±319	1796 ±341		P<0.001
	Range	10-62	3-161	23-434	8-970	122-1462	255-1554	513-2099	792-3411	1777-2414	835-3161		
Testoste-rone (nmol)	N	19	89	158	152	72	36	14	12	3	9	3	566
	M±SEM	5 ±1	7 ±1	15 ±1	28 ±2	51 ±4	67 ±8	88 ±16	182 ±31	179 ±34	159 ±44	171± 103	P<0.001
	Range	1-28	1-33	2-61	2-103	5-215	4-179	26-272	29-357	114-231	47-377	40-374	

Data is mean ±SEM. P-value <0.05 considered significant (ANOVA test).
