# Supplementary material for: Hallmarks of Human Small Antral Follicle Development: Implications for Regulation of Ovarian Steroidogenesis and Selection of the Dominant Follicle
Source: Front Endocrinol (Lausanne). 2018 Jan 12;8:376. doi: 10.3389/fendo.2017.00376 (PMC5770355; doi:10.3389/fendo.2017.00376)
Supplement: Supplementary file 3 [file Table_3.PDF]

Supplemental table 3. mRNA gene expression of granulosa cell specific substances in human small antral follicles in relations to follicular diameter. Data is expressed in relation to the corresponding *GAPDH* mRNA expression.

		FOLLICULAR DIAMETER (MM)									
**Gene**		**<3.5**	**3.5-4.5**	**4.5-5.5**	**5.5-6.5**	**6.5-7.5**	**7.5-8.5**	**8.5-9.5**	**9.5-10.5**	**10.5 – >12.5**	**SUM No. /P-value**
FSHR	**N**	9	47	85	88	43	21	10	7	5	315
	**M±SEM**	801 ±531	372 ±87	218 ±36	175 ±22	229 ±39	139 ±34	108 ±48	187 ±69	114 ±99	P<0.002
	**Range**	35-4924	1-2440	1-2355	1-947	1-1016	1-476	4-459	13-417	1-508	
CYP19a1	**N**	9	33	66	79	33	16	6	7	3	252
	**M±SEM**	46 ±20	58 ±17	107 ±24	92 ±83	62 ±31	74 ±40	29 ±11	172 ±130	326 ±288	P>0.10
	**Range**	1-203	1-424	2-969	1-846	1-402	3-634	9-68	4-945	4-901	
AR	**N**	12	49	84	87	45	17	10	7	5	316
	**M±SEM**	260 ±106	235 ±68	137 ±22	83 ±11	116 ±35	67 ±16	36 ±12	80 ±38	62 ±36	P<0.03
	**Range**	27-1217	1-3031	1-1369	1-505	1-1180	12-274	4-114	3-283	3-187	
AMHR2	**N**	6	27	54	61	28	10	7	4	4	201
	**M±SEM**	9 ±2	12 ±2	20 ±4	17 ±2	19 ±4	14 ±3	11 ±4	10 ±5	21 ±12	P>0.10
	**Range**	4-16	2-30	1-196	1-54	0.1-106	1-26	0.1-32	1-23	5-55	
AMH	**N**	13	40	74	74	39	19	8	6	4	277
	**M±SEM**	4021 ±1974	1425 ±307	1973 ±421	1347 ±261	1520 ±524	668 ±289	532 ±410	1094 ±639	324 ±318	P>0.10
	**Range**	188-24900	1.5-7622	0.01-18220	0.1-14900	0.3-19750	2.2-5410	1.5-3325	11.8-3222	0.1-1279	

Data is mean ±SEM. P-value <0.05 considered significant (ANOVA test).
